# Gold Nanoparticles Disrupt Mitochondrial Activity in Hypothalamic POMC Cells: Implications for Energy Homeostasis

**DOI:** 10.3390/nano15161291

**Published:** 2025-08-21

**Authors:** Boglárka Mária Schilling-Tóth, Silvia Ondrašovičová, Eszter Vámos, Balázs Radnai, Daiana Alymbaeva, Tibor Bartha, István Tóth, Dávid Sándor Kiss

**Affiliations:** 1 Department of Physiology and Biochemistry, University of Veterinary Medicine, H-1078 Budapest, Hungary; schilling-toth.boglarka.maria@univet.hu (B.M.S.-T.); daiana.alymbaeva@univet.hu (D.A.); bartha.tibor@univet.hu (T.B.); kiss.david@univet.hu (D.S.K.); 2 Department of Biology and Physiology, University of Veterinary Medicine and Pharmacy in Košice, 041 81 Košice, Slovakia; silvia.ondrasovicova@uvlf.sk; 3 Department of Biochemistry and Medical Chemistry, University of Pecs Medical School, H-7601 Pécs, Hungary; eszter.vamos@aok.pte.hu (E.V.); balazs.radnai@aok.pte.hu (B.R.)

**Keywords:** oxygen consumption rate, mitochondrial activity, melanocortin system, AuNP, Seahorse XF analyzer, energy metabolism, nanoparticle toxicity

## Abstract

**Background:** Gold nanoparticles (AuNPs) have several beneficial properties that make them effective as intracellular drug carriers, and their potential for various diagnostic and therapeutic applications is gaining recognition. Depending on their size and shape, AuNPs can cross the central nervous system (CNS) through the blood–brain barrier (BBB). In the CNS, they can exert a variety of influences on neuronal and glial cells, which can be both supportive—promoting cell health and function—and cytotoxic, potentially leading to cellular damage. The hypothalamus (HT) is the first region where nanoparticles (NPs) interact, as this neuroendocrine center is particularly sensitive to factors in the systemic circulation due to its function and location. This area is affected by systemic factors, including pro-opiomelanocortin (POMC) neurons, which regulate metabolic function and maintain homeostasis. The activity of mitochondria within these cells influences their response to both external factors and the presence of AuNPs, thereby facilitating a complex interplay between nanoparticle interactions and cellular metabolism in this vital brain region. **Aims:** This study investigates how AuNPs, at different concentrations and exposure times under in vitro conditions, affect the mitochondrial activity of POMC neurons, aiming to provide a comprehensive understanding of the mechanisms in the HT. **Methods**: The study investigates the effect of varying gold nanoparticle concentrations on the mitochondrial activity of POMC neurons over treatment periods of 1, 15, 24, and 48 h. Mitochondrial activity was measured using a Seahorse XFp Analyzer to provide high-resolution insights. Additionally, mitochondrial functionality was assessed through the detection of reactive oxygen species (ROS) and cell viability. **Results:** The findings indicated that the effects of gold nanoparticles on mitochondrial activity depend significantly on their concentration and exposure time. Specifically, exposure leads to an increase in early response systems, the citric acid cycle, and proton efflux, ultimately resulting in the inhibition of mitochondrial function and ATP production in POMC cells. This disruption may affect hypothalamic regulation and energy metabolism.

## 1. Introduction

Over the past fifteen years, nanoparticles (NPs) have considerably transformed diagnostic and therapeutic medicine methodologies [1]. One member of the NP family, the gold nanoparticles (AuNPs), due to their high stability, low toxicity, and relative ease of synthesis, have been distinguished as an option for several medical applications [2]. As drug carriers, AuNPs have a high affinity for conjugating with proteins and other biomolecules, maintaining their functionality and their biological activity; AuNPs play a role in targeted drug delivery systems in therapeutics [3,4,5,6]. Not only can these NPs bind multiple drugs, but they can also serve as carriers for gene therapy, enabling site-specific treatments with reduced systemic effects [7,8]. They also act as adjuvants to enhance immune responses [9]. These are all advantages when NPs are used as a drug or carrier system. The effect depends on the size, shape, charge, and surface modifications of AuNPs, which strongly influence their biodistribution, cellular uptake, and excretion [10,11,12,13]. The shape and origin of the AuNPs enhance their effectiveness while minimizing potential side effects [14,15].

The use of AuNPs is considered a promising approach in cancer treatment because of their ability to deliver various chemotherapy agents directly to tumor tissues [16,17]. This application has also contributed to the development of new treatment options in oncology, such as photothermal therapy. In this process, AuNPs absorb infrared light and convert it into heat, enabling targeted destruction of tumor cells while minimizing damage to nearby healthy tissues [18]. For inflammatory conditions, AuNPs with functional coatings, conjugation, or biosynthetic methods—where bioactive plant compounds reduce and stabilize metal ions during nanoparticle production—are increasingly used due to their antioxidant properties and ability to reduce inflammatory cytokines and reactive oxygen species (ROS) [19,20,21]. Because of their small size, AuNPs can cross the blood–brain barrier (BBB), and their potential anti-neuroinflammatory effects suggest they could be employed for treating neurological disorders such as Alzheimer’s and Parkinson’s diseases [22]. However, the effects of specific NPs on the central nervous system (CNS) are still not fully understood, requiring further high-resolution research [23,24]. Particles measuring 15–20 nm are particularly effective at crossing the BBB, while larger particles (>50 nm) tend to accumulate mainly in the liver and spleen [10,17,21]. Generally, AuNPs smaller than 50 nm with a spherical shape show optimal distribution across tissues, including the BBB [17,24,25]. These particles can also influence BBB permeability by altering the structure and function of tight junction proteins, potentially allowing active compounds or pathogens to enter the brain [26]. In clinical settings, NPs have received FDA approval for treating various medical conditions, including ovarian, breast, prostate, and lung cancer, as well as leukemia, Crohn’s disease, rheumatoid arthritis, psoriatic arthritis, and ankylosing spondylitis [27] (e.g., NCT03815916, NCT03993171, NCT04098406). Despite this limitation, NPs are being explored for brain targeting in preclinical models and ongoing clinical trials, likely due to the controversial effects of NPs on the brain. The effects of NPs on neuronal activity exhibit considerable variation, influenced by their distinct material properties. Some studies suggest that AuNPs can enhance neuronal activity through several mechanisms, including increasing the frequency of action potentials, raising the input resistance of neurons, lowering the threshold and duration of action potentials, and shortening the post-action potential hyperpolarization period, while increasing reactive species and causing inflammation in the brain, depending on their size and shape [22,28]. Several studies have shown that AuNPs can enhance neural differentiation. However, the most notable effects have been observed in neurodegenerative diseases, where the administration of AuNPs is considered as a potential treatment. These diseases are associated with hypertension, hyperglycemia, dyslipidemia, chronic low-grade inflammation, and mitochondrial dysfunction [22]. Several studies suggest that incorporating AuNPs may have a positive effect on metabolic disorders, and these controversial effects may also be relevant to neurodegenerative diseases [29]. Therefore, it is highly necessary that we discover the insights into these controversial effects and their influence on the main metabolic processes regarding the mitochondria.

The hypothalamus is a vital brain region responsible for maintaining homeostasis by regulating autonomic functions, hormones, circadian cycles, metabolism, and behavior. Unlike many parts of the brain, the hypothalamus (HT) has areas where the blood–brain barrier (BBB) is more permeable, especially around the median eminence and the arcuate nucleus (ARC). This structural trait allows the HT to directly interact with circulating hormones and systemic signals, making it especially vulnerable to external influences like nanoparticles [30].

Given the increased permeability and the HT’s proximity to systemic circulation, it is likely that AuNPs could target this area soon after entering the bloodstream. However, our current understanding of how AuNPs influence the melanocortin system remains limited. Clarifying this relationship is a crucial step in understanding their overall effect on homeostasis and metabolic regulation at the organism level. Notably, AuNPs may accumulate in the HT because of its higher permeability. The ARC, which contains melanocortin neurons including proopiomelanocortin (POMC) cells, is essential for maintaining energy balance and homeostasis. POMC neurons promote catabolism and satiety through the anorexigenic actions of α-Melanocorticoid Stimulating Hormone (MSH) [31,32].

AuNP uptake into cells occurs through various mechanisms, including phagocytosis and clathrin- or caveolae-dependent endocytosis [33]. Smaller, spherical particles that are slightly negatively or neutrally charged are internalized more efficiently [33,34]. Once inside the cell, AuNPs can localize to different organelles, such as endosomes, lysosomes, and especially mitochondria. Mitochondrial metabolism adjusts to meet the energy needs of the host cell. This process is particularly specialized in melanocortin neurons, known as POMC cells, which act as regulatory neurons that integrate peripheral signals with central nervous system inputs [31]. In these cells, mitochondrial activity functions as a “homeostatic sensor”, translating systemic cues into cellular responses. Notably, mitochondrial dynamics in POMC neurons are highly sensitive to circulating signals, which fine-tune the regulatory processes driven by the melanocortin system itself. Therefore, studying mitochondrial function in these neurons is crucial for understanding homeostasis at the organism level [35]. The impact of AuNPs on mitochondria is not fully understood. Existing studies report varying effects depending on particle size, shape, and surface modifications. Among their adverse effects, AuNPs have been shown to induce mitochondrial stress, increase ROS production, and compromise membrane potential and structural integrity. These properties are highly relevant in POMC neurons, where mitochondrial dynamics are tightly linked to the regulatory processes of the melanocortin system. Despite their significance, little is known about how AuNPs affect the mitochondria of these neurons; exploring these interactions could yield valuable insights into their neuronal and mitochondrial effects and their systemic impact, where the POMC cells can be the first step to understand how the initial effect contributes to the diseases [31,35].

Our study hypothesized that AuNPs reaching the HT cell through the BBB can alter hypothalamic regulatory processes and, consequently, systemic metabolism and homeostasis, at least in part by affecting the mitochondrial activity of POMC neurons. To test this hypothesis, this study aims to investigate the effects of 20 nm spherical AuNPs, commonly used as a foundational compound in many NP preparations, on the mitochondrial activity of POMC neurons under in vitro conditions, specifically evaluating mitochondrial activity (i.e., respiration rates) in response to varying AuNP concentrations and exposure durations.

## 2. Materials and Methods

### 2.1. Cell Line

The studies were performed on immortalized POMC cell cultures isolated from mice (mHypoA-POMC/GFP-1-4; Cedarlane Laboratories, Burlington, ON, Canada).

The cell cultures were maintained in Dulbecco’s Modified Eagle Medium (DMEM, Merck, Darmstadt, Germany, cat. no: P4333), supplemented with 10% Fetal Bovine Serum (FBS, Merck, cat. no: F7524), and 1% penicillin–streptomycin solution (P/S, Merck, cat. no: P4333) in a CO_2_ incubator (37 °C, 5% CO_2_). After thawing, the cells were maintained in T75 cell flasks until 90% confluency, then harvested using a 0.25% trypsin-PBS (Merck, cat. no. BE17-160E-T4049) solution, counted, and their viability measured. They were then seeded for subsequent experiments.

### 2.2. Treatment

Spherical 18–22 nm AuNPs (Merck, cat. no: 741965) were applied in three concentrations: 7 pM, 7 nM, and 7 µM in DMEM, which was previously described to affect cell viability [36]. The measurements were performed after 1, 15, 24, and 48 h incubation periods to describe the direct and long-term effects.

### 2.3. Seahorse Measurement

The Seahorse XF Analyzer (Agilent Technologies, Santa Clara, CA, USA) measures the oxygen consumption rate (OCR), which reflects mitochondrial respiration, and the extracellular acidification rate (ECAR), which indicates glycolytic activity. By introducing specific inhibitors and uncouplers during the assay, researchers can analyze the contributions of different mitochondrial complexes and processes to overall respiration, enhancing our understanding of mitochondrial function, dysfunction, and bioenergetics.

Mitochondrial respiration relies on the Mitochondrial Electron Transport Chain (ETC), a series of protein complexes located in the inner mitochondrial membrane. During measurements with the Seahorse XF Analyzer, various compounds are sequentially injected to assess different aspects of mitochondrial function. For instance, Oligomycin (OM) inhibits ATP synthase activity, while Carbonyl Cyanide-p-trifluoromethoxy-phenylhydrazone (FCCP) acts as an uncoupler, disrupting proton gradients. Rotenone (RotA) inhibits Complex I, and Actinomycin (AM) targets Complex III.

**Figure 1:**
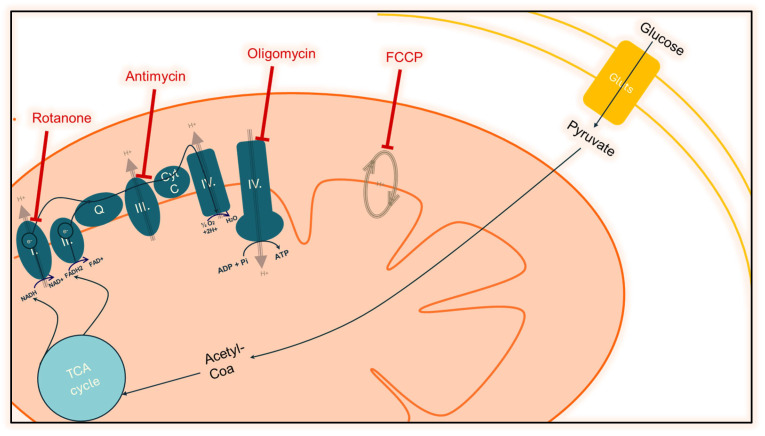
Schematic figure of the Seahorse XF Mito Stress solution’s inhibitor effect on the mitochondrial respiratory chain.

These inhibitors allow the assay to quantify multiple facets of mitochondrial function. Basal respiration is measured under standard conditions before any drugs are added. After the injection of Oligomycin, ATP-linked respiration can be assessed since ATP synthase activity is inhibited. Following FCCP injection, the maximal respiration capacity can be determined, while the spare respiratory capacity is evaluated with RotA. Additionally, after adding AM, the proton leak and residual respiration can be measured by blocking Complexes I and III. Together, these measurements facilitate the detection of AuNPs’ respiratory effects and provide insight into the mechanisms at play. From this high-resolution analysis the following additional results can be estimated: the ATP production from the basal minus the ATP-linked oxygen consumption mean values, the residual respiration and the proton leak from the basal minus the OCR after blocking the I and III Complexes (Figure 1).

The Mito Stress assay was carried out according to the manufacturer’s instructions: the required Agilent materials (Seahorse assay), in Agilent Seahorse XFp cartridges (Agilent Seahorse FluxPak, Santa Clara, CA, USA), using the Agilent Seahorse XF Cell Mito Stress Test Kit (Agilent, Santa Clara, CA USA, cat. no: 103010-100).

The Agilent Seahorse Extracellular Flux XFp Analyzer (Agilent Technologies, Santa Clara, CA, USA) was utilized to measure the OCR of POMC cells, following a previously established protocol [37]. The sensor cartridges were hydrated in XF calibrant and maintained at 37 °C without CO_2_ overnight. POMC cells were plated in XF Miniplates at a density of 3 × 10^4^ cells in 80 μL of complete growth medium (DMEM for Primary Cell Isolation) and incubated at 37 °C with 5% CO_2_ for two days. The Agilent Seahorse XF Base Medium, containing 1 mM pyruvate, 2 mM glutamine, and 10 mM glucose (adjusted to a pH of 7.4 with 0.1 N NaOH), replaced the Primary Cell Isolation medium on the cells.

Before measurements, various compounds were added to the appropriate ports of the sensor cartridge: 10 μM Oligomycin, 10 μM FCCP, and 5 μM rotenone/antimycin. Three parallel measurements (n = 3) were conducted for each treatment. The Seahorse XF technology automatically measures the OCR, which reflects mitochondrial respiration, and the ECAR, where the flux of protons indicates glycolysis [38].

### 2.4. Viability Test

Cell viability was assessed using the Tox8 resazurine-based in vitro toxicology assay (Merck, cat. no. Tox8-1KT), which was previously described to reflect mitochondrial respiration closely by measuring the metabolic activity of cells, specifically through the conversion of resazurin to resorufin. This process is indicative of mitochondrial respiration and the function of the TCA cycle [39,40]. For cell maintenance, the same culture medium was used as in the Seahorse experiments. One day prior to treatment, 104 POMC cells were seeded into each well of a 96-well plate and incubated overnight in a CO_2_ incubator at 37 °C. On the day of the treatment, the growth medium was changed to DMEM and treated with three different concentrations (7 pM, 7 nM, and 7 µM) of AuNPs and measured after 1, 15, 24, and 48 h incubation to refer to the Seahoarse measurements [40]. The fluorescence was measured after 2 h of adding the Tox8 resazurine-based assay reagent by using a SpectraMax iD3 microplate reader (Molecular Devices, San Jose, CA, USA) in fluorescence mode (excitation: 560 nm; emission: 600 nm). Six parallel measurements were performed for all treatments, with no treatment for the positive control, and 10% Tween-20 in DMEM as a negative control.

### 2.5. ROS Assay

ROS was assessed using the (Merck, cat. no. MAK-143), an intracellular ROS kit. As recommended by the manufacturer, 104 POMC cells per well were seeded into a 96-well plate and incubated in the CO_2_ incubator overnight before the measurements. For cell maintenance, the same culture medium and treatment medium was used as in the Seahorse experiments. The fluorescence was measured using a SpectraMax iD3 microplate reader (Molecular Devices) in fluorescence mode (excitation: 490 nm; emission: 530 nm). Six parallel measurements were performed for all treatments, with no treatment in the negative control, and 10 µM H_2_O_2_ solutions in DMEM as the positive control.

### 2.6. Data Analysis

These tests were performed using GraphPad Prism version 9 (GraphPad Software, San Diego, CA, USA) and with the Department of Biostatistics at the University of Veterinary Medicine, Budapest, Hungary. The OCR values were divided by the cell number for data analysis [41]. Following the determination of normality of the distribution, statistical analyses were performed using one-way or two-way ANOVA, and for multiple comparisons, the Sῐrak test was used. *p*-values less than 0.05 (*p* < 0.05) were considered statistically significant.

## 3. Results

### 3.1. Seahorse XFp Measurements

**Figure 2:**
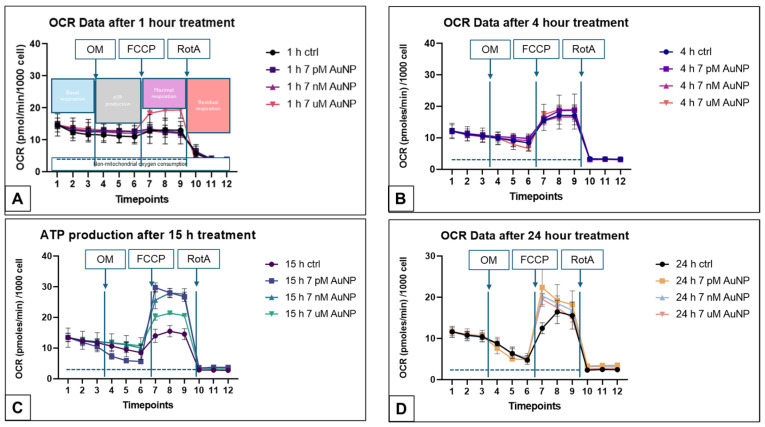
Oxygen consumption rates (OCR) in POMC cells under various AuNP treatments and incubation time in Seahoarse XF MitoStress experiments (n = 3, Mean ± SD). The administration of the 12 Timepoints is generated automatically, measuring the basal respiration (Timepoints 1–3), the ATP-linked respiration (Timepoints 4–6) after Oligomycin (OM) treatment, the maximal respiration (Timepoints 7–9) after Carbonyl Cyanide-p-trifluoromethoxy-phenylhydrazone (FCCP) treatment, and the residual respiration (Timepoints 10–12) after Rotamin Antimycin (RotA) treatment. (**A**): OCR data after 1 h of 0 (ctrl), 7 pM, 7 nM, and 7 µM AuNP treatment. (**B**): OCR data after 15 h of 0 (ctrl), 7 pM, 7 nM, and 7 µM AuNP treatment. (**C**): OCR data after 24 h of 0 (ctrl), 7 pM, 7 nM, and 7 µM AuNP treatment on POMC cells. (**D**): OCR data after 48 h of 0 (ctrl), 7 pM, 7 nM, and 7 µM AuNP treatment on POMC cells.

To assess mitochondrial function under the specified experimental conditions, we primarily utilized oxygen consumption rates (OCRs), as this measure most accurately reflects mitochondrial activity. The second figure presents the OCR data relative to cell number. Seahorse measurements record basal respiration, ATP-linked respiration, maximal respiration, and residual respiration in real-time at three different points. Three replicate measurements were performed for each incubation time and concentration. Every treatment concentration influenced the OCR, which was observed at different concentrations, dependent on the incubation time (Figure 2).

We did not find a significant alteration in the basal or the ATP-linked OCR data. However, after 15 h, a tendency of decrease in the ATP-linked respiration could be measured, but it was not statistically significant (Figure 2C).

**Figure 3:**
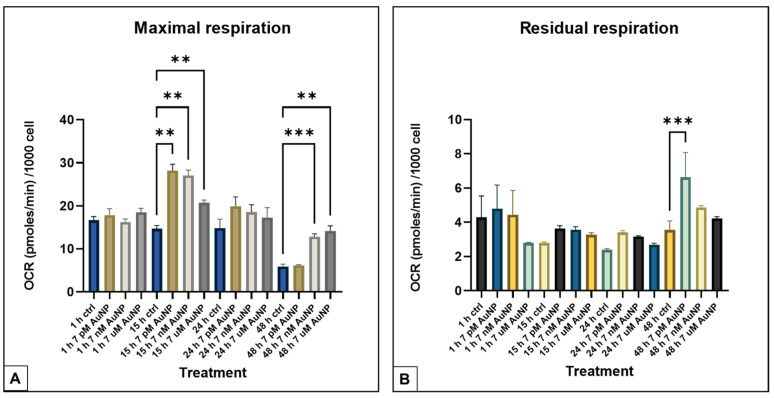
OCR (pmole/min) normalized to the cell number in POMC cells under various AuNP treatments and incubation times. (**A**). Maximal respiration: the figure displays OCR values during Carbonyl Cyanide-p-trifluoromethoxy-phenylhydrazone (FCCP) induced maximal respiration (mean ± SD) at different incubation times (1 h, 4 h, 15 h, and 24 h) with different AuNP treatment concentrations 7 pM, 7 nM, and 7µM (n = 3). (**B**). Residual respiration: the figure displays OCR values during Rotenone/Antimycin induced residual respiration (mean ± SD) at different incubation times (1 h, 4 h, 15 h, and 24 h) with different AuNP treatment concentrations 7 pM, 7 nM, and 7µM (n = 3). All OCR values are normalized to their cell number. Statistically significant differences. ** *p* < 0.01, *** *p* < 0.001.

The OCR data showed an increase in maximal respiration after 15 h of treatment at every concentration of the AuNPs (7 pM, 7 nM, and 7µM). In that time, the effect decreased in the lowest concentration (7 pM at 48 h) (Figure 3A). Even though the maximal respiration elevation diminished at the lowest concentration of the treatment, the residual respiration elevated at the same time (Figure 3B).

**Figure 4:**
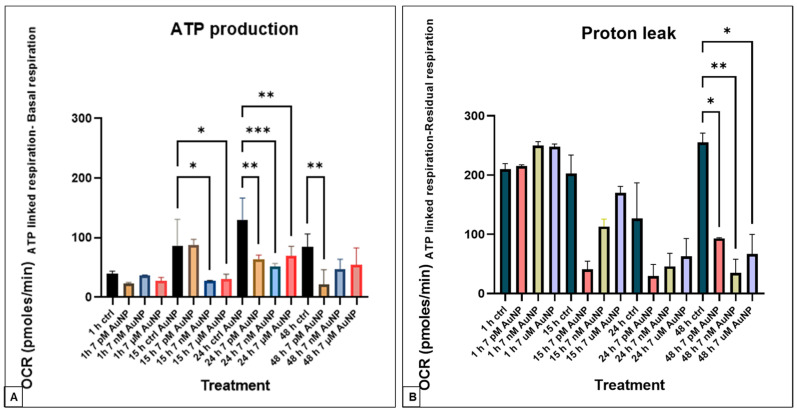
(**A**). **ATP production in the POMC cells.** AuNPs’ effect after 7 pM, 7 nM, and 7 µM AuNP treatment at the following incubation times: 1 h, 15 h, 24 h, and 48 h. The ATP production was calculated from the OCR levels, ATP-linked respiration, and basal respiration (n = 3, Mean ± SD). (**B**). **Proton leak in the POMC cells.** AuNPs’ effect after 7 pM, 7 nM, and 7 µM AuNP treatment at the following incubation times: 1 h, 15 h, 24 h, and 48 h. The proton leak was calculated from the OCR levels, ATP-linked respiration, and residual respiration (n = 3, mean ± SD). Statistically significant differences are * *p* < 0.05, ** *p* < 0.01, *** *p* < 0.001.

The assessmnt of ATP production showed no significant changes within the 1-h treatment groups, especially regarding maximal respiration. However, notable differences appeared in the 15-h treatment groups. Specifically, a significant decrease in oxygen consumption rate (OCR) was observed at the mid- (7 pM) and highest (7 µM) concentrations after 15 and 24 h of AuNP exposure. Conversely, at the lowest concentration, the inhibitory effects on ATP production were initially delayed. However, they lasted up to 48 h, after which the differences were no longer statistically significant in the other groups. These results indicate that AuNPs’ effect on ATP production depends on both time and concentration, with significant reductions emerging after more extended incubation periods and at lower concentrations (Figure 4A). We measured a significant reduction in the proton leak after 48 h (Figure 4B).

### 3.2. Cytotoxicity Assay

**Figure 5:**
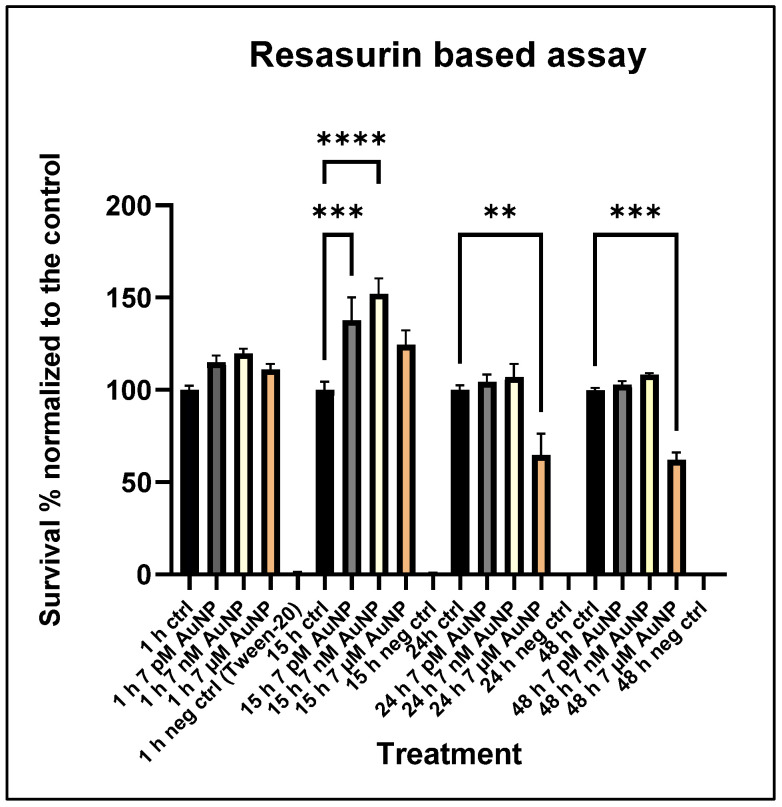
**Viability measurements using the Tox8 assay**. POMC cell viability after 7 pM, 7 nM, and 7 µM AuNP treatment at the following incubation times: 1 h, 15 h, 24 h, and 48 h. The OCR values (n = 6, Mean ± SE). Viability was normalized to the mean of the untreated control group (100%) and the negative control group (Tween-20, 0%). Statistically significant differences are ** *p* < 0.01, *** *p* < 0.001, **** *p* < 0.0001.

To evaluate the overall toxicity of AuNPs, cell viability was performed using the Tox8 resazurin-based in vitro toxicology assay. These cytotoxicity assay measurements indicated that AuNP treatments have a significant effect on cell viability across the treatment groups compared to controls. Initially, there was an increase in cell viability in the mid- and low concentration treatments (7 nM, 7 pM) at 15 h, particularly related to the TCA cycle, but this effect diminished over time. The highest concentration of AuNPs caused toxic effects, including TCA cycle arrest, at later stages in POMC cells, which showed a late effect after 24 and 48 h of incubation time (Figure 5).

### 3.3. ROS Measurements

**Figure 6:**
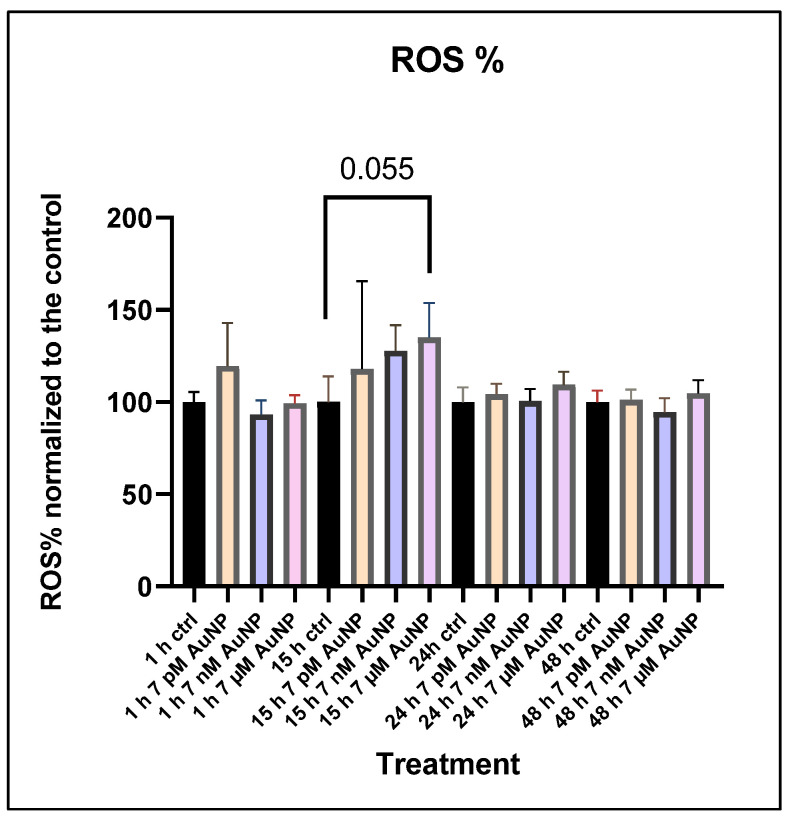
**ROS measurement using the intracellular ROS detection kit.** POMC cell viability after 7 pM, 7 nM, and 7 µM AuNP treatment at the following incubation times: 1 h, 15 h, 24 h, and 48 h. The OCR values (n = 5, Mean ± SE). Viability was normalized to the mean of the untreated control group (100%). Statistically significant differences are * *p* < 0.05.

After measuring cytotoxicity and mitochondrial alteration, we aimed to identify the main source of the cytotoxic effect and mitochondrial disturbance. Therefore, we measured the percentage of intracellular ROS produced after AuNP treatment. The results showed an increase after treatment, but only at the highest concentration (7 µM) was this level close to a significant increase in POMC cells (Figure 6).

## 4. Discussion

Our study’s findings indicate that nanoparticles (NPs) exert distinct effects on various aspects of mitochondrial respiration in POMC cells. We measured an increase in the maximal respiration rate. At the same time, the oxygen consumption rate, associated with ATP production, showed a significant decrease compared to the control, with the extent of the effect depending on the treatment concentration and incubation time. These findings indicate a selective effect of NPs on mitochondrial processes, particularly those related to energy generation, and may provide insight into the contradiction in the effect on neural cells.

We observed both an early and a later effect of the NPs, depending on the treatment concentration and incubation time. In the early effect, we saw an increase in the activation of the TCA cycle, indicating higher cell viability. Although TCA cycle activity also increased, a decrease in ATP-linked oxygen consumption suggests a possible disruption in the connection between proton flow and the ATP synthase enzyme. Specifically, protons might be unable to return from the intermembrane space to the matrix via ATP synthase, which would hinder efficient ATP production. This idea aligns with a similar effect seen during senescence-associated changes, where the disturbance initially causes increased mitochondrial activity, followed by a decline in ATP production and a mitochondrial stress response [42]. However, we could not detect a significant increase in ROS production, suggesting that the effect occurs through different pathways rather than via ROS-induced oxidative stress or antioxidant incapacity.

Similarly, silver NPs have been shown to inhibit ATP synthase activity in mitochondria isolated from rat liver cells [43]. However, the observed effects can be significantly influenced or even reversed by NP’s size, surface coatings, or conjugates [27,28]. For example, 13 nm AuNPs coated with 11-mercaptoundecanoic acid enhanced ATP production and, at high concentrations, reduced mitochondrial membrane potential in kidney cell cultures. In contrast, no such effects were observed with 60 nm AuNPs coated with sodium citrate [44,45], and in a separate study examining the combined impact of ionizing radiation and treatment with AuNPs during irradiation, there was a notably increased OCR during basal respiration. The discrepancy with our findings is likely due to the use of much smaller, 1.9 nm particles in that study [46].

Furthermore, NPs could also decrease ATP production by disrupting the supply of essential substrates for ATP synthesis. AuNPs can reduce the levels of end products of the TCA cycle (e.g., NADH, FADH2), which we could observe after 15 h of incubation. This effect was also dependent on concentration [47]. This cycle inhibition results in fewer cofactors entering the electron transport chain, thereby reducing electron flow and proton pumping, which in turn lowers the proton gradient and subsequently decreases ATP synthesis. Our findings on maximal respiratory capacity support this idea. While stimulating changes were observed in cells after 15 h, a significant decrease in ATP production and cytotoxic effects occurred across all three concentrations after 15 h of exposure, at which point the cells began to divide and respond to the stress effect on the mitochondrial changes. This indicates not only a selective effect of several AuNP concentrations but also a cumulative effect of NPs over time, potentially impairing the mitochondria’s ability to produce ATP, but not the proton gradient. Such progressive damage to mitochondrial activity could have long-term effects, consistent with previous research on the time-dependent localization of AuNPs within cells [48,49]. While the latter effect showed that ATP production returned to the normal level, the elevation of residual respiration indicates that the disturbance in mitochondrial respiration remains at the lowest level following the AuNP treatment [50,51].

NP treatment is known to affect ROS production in different ways. While AuNPs can decrease obesity-related ROS levels and boost antioxidant enzyme activity, they may also increase ROS, leading to mitochondrial damage that could reduce OCR parameters [30,52]. This dual role of ROS reflects their complex part in mitochondrial functions. ROS can both cause and result from mitochondrial dysfunction, creating a feedback loop that worsens oxidative stress [53]. We did not observe ATP production arrest triggered through this mechanism; intracellular ROS levels were not significantly higher than in the control group. On the other hand, AuNPs may influence mitochondrial biogenesis by altering the number, size, and ultrastructure of mitochondria through processes such as fusion and fission [54]. Disrupting the fusion–fission balance, as seen with silica NPs [55], could cause mitochondrial morphological changes and impair their function. The buildup and aggregation of AuNPs inside mitochondria could further worsen these effects by causing mechanical stress and deformation.

**Figure 7:**
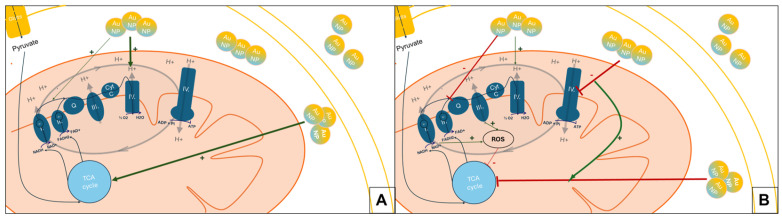
**Early and late responses of the AuNPs on mitochondrial respiration.** (**A**) **Early responses** caused an increase in viability (TCA cycle), proton efflux, and ETC. (**B**) **Late responses** caused increased toxicity and lower levels of ROS, arrest in ATP production, and ETC while the proton efflux was reduced.

In conclusion, the observed effects of AuNPs on mitochondria are influenced by multiple factors, including particle size, surface chemistry, exposure duration, and ROS dynamics. Further research is necessary to clarify the mechanisms behind these effects and their implications for cellular and organismal health. In Figure 7, we summarize the early and later effects of the nanoparticles, based on our findings.

Although AuNPs are detected in the CNS after crossing the BBB and can also be detected in the hypothalamus [56], their impact on hypothalamic processes, and consequently on the organism’s metabolism, remains a relatively underexplored area. Additionally, there is limited information on how these NPs affect the organism’s metabolic processes.

Studies examining the metabolic effects of AuNPs often focus on biosynthetically produced NPs, analyzing their influence on metabolism rather than their direct effects on hypothalamic regulatory processes. The impact of AuNPs on the ARC has been studied in various contexts, including reproductive processes, among the primary functions regulated by this brain area. An in vivo experiment demonstrated that 8–12 nm AuNPs could inhibit the activity of the hypothalamic–pituitary–gonadal axis by reaching the hypothalamus and reducing the size of the nuclei in the arcuate nucleus and preoptic nucleus. Interestingly, some studies show that silver nanoparticles (AgNPs) and silver ions (Ag^+^) similarly reduce the size of the nuclei in these two brain areas, with a more pronounced effect than gold nanoparticles [57,58]

As mentioned above, mitochondria in POMC cells of the hypothalamus function as critical energy sensors, integrating peripheral metabolic signals with cellular energy demands. These organelles regulate ATP production and ROS levels, enabling POMC neurons to respond to fluctuations in systemic energy availability. By acting as metabolic hubs, mitochondria in POMC cells play a pivotal role in controlling neuronal activity and transmitting signals that influence whole-body energy homeostasis [59]. Our current experiment revealed a pronounced time-dependent decrease in mitochondrial activity, rather than a concentration-dependent one, in POMC cells. Reduced ATP production is suggested to diminish the ability of POMC neurons to respond to metabolic and hormonal signals, which might be manifested in reduced production and release of α-MSH, among others [31]. Higher maximal respiratory capacity suggests compromised mitochondrial flexibility and resilience, limiting the ability of POMC neurons to adapt to sudden changes in energy demand [60] while mediating the ETC [61], which can lead to cytotoxic effects. This could impair their functionality as “energy sensors” [31]. Together, these effects may weaken the activity of POMC neurons, reducing their output of anorexigenic (appetite-suppressing) signals. This could result in hyperphagia (increased food intake) and reduced energy expenditure, disrupting energy balance and potentially contributing to weight gain and metabolic dysregulation. Over time, these changes can exacerbate conditions such as obesity, insulin resistance, and other metabolic disorders, ultimately undermining the body’s ability to maintain energy homeostasis. Compared to our results, a previous in vivo experiment indicated that AuNPs inhibited overeating and obesity induced by the antipsychotic olanzapine by increasing POMC cell protein expression, suggesting an effect similar to our early effect results [62]. This discrepancy could stem from differences in NPs’ size (2 nm particles were used in the experiment), shape (nanoclusters in this research), and the complexity of in vivo processes at the organism level versus our in vitro findings.

Beyond mitochondrial activity, the ROS levels in melanocortin system (MS) cells profoundly influence the organism’s metabolism. POMC neurons sense a positive energy state through increased ROS levels generated by glucose oxidation, which enhances POMC neuron firing and suppresses food intake. Conversely, negative energy balance (low glucose levels) is associated with increased ROS levels. Thus, ROS levels play a critical role in regulating hypothalamic neuron function and systemic metabolism [63]. As reported in the literature, maximal respiration reflects cellular stress responses and mitochondrial quantity, with increased mitochondrial respiration often indicating stress. At elevated ROS levels, cells may undergo mitochondrial biogenesis or fission to compensate for stress, potentially amplifying ROS production [64]. In our experiment, we found that this dysfunction was not caused by ROS production, but by the arrest of ATP production and proton leak. A significant increase in maximal respiration was observed in groups treated for 15 and 48 h, suggesting that shorter AuNP treatments did not generate sufficient arrest to induce detectable cellular stress responses, but a cytotoxic energy decrease, which was time and dose dependent. Therefore, future experiments should focus on fusion–fission, senescence-associated secretory phenotype (SASP), neuronal firing activity, and ultrastructural changes (including mitochondrial dynamics) in these neurons, alongside respiratory parameters. However, the effects of POMC cell alterations on the whole organism cannot be fully understood without considering other components of the melanocortin system, such as their counterpart, AgRP neurons.

## 5. Conclusions

In conclusion, our in vitro findings, supported by prior research, suggest that 20 nm spherical AuNPs without active ingredients or coatings inhibit mitochondrial activity in POMC cells. Early on, the TCA cycle and proton gradient efflux, based on Complex activities, showed an increasing effect. Later, we observed inhibition, evidenced by reduced ATP production after 15, 24, and 48 h of incubation, while higher doses returned to normal levels and also caused cell death. We saw a dose-dependent disruption of the mitochondrial proton gradient only after 15 h, indicating a cumulative effect. By impairing mitochondrial activity in POMC cells, AuNPs may alter hypothalamic regulatory processes, potentially disrupting energy homeostasis. This could lead to reduced satiety, increased food intake, hyperphagia, weight gain, and metabolic disorders. Further studies are needed to confirm these effects.

## Data Availability

The University of Veterinary Medicine Budapest (UVMB), where the measurements were carried out, Hungary (UVMB) follows the FAIR principles guidelines, which are a set of guidelines to enhance the usability and value of digital assets (data, software, etc.) by making them findable, accessible, interoperable, and reusable. All results are compiled and stored in the UVMB research group’s repository.

## Abbreviations

The following abbreviations are used in this manuscript:

AuNPs	Gold Nanoparticles
CNS	Central Nervous System
FDA	Food and Drug Administration, USA
HT	Hypothalamus
POMC	Pro-Opiomelanocortin
ROS	Reactive Oxygen Species
NPs	Nanoparticles
BBB	Blood–Brain Barrier
ARC	Arcuate Nucleus
MSH	Melatonin Stimulating Hormone
DMEM	Dulbecco’s Modified Eagle Medium
FBS	Fetal Bovine Serum
P/S	Penicillin Streptomycin
OCR	Oxygen Consumption Rate
ECAR	Extracellular acidification rate
OM	Oligomycin
FCCP	Carbonyl Cyanide-p-trifluoromethoxy-phenylhydrazone
Rot	Rotanon
AM	Animycin
TCA	Tricarboxylic Acid Cycle
RotA	Rotanon/Antimycin
